# Potential of *Beauveria* Species Isolated from Southern Kazakhstan for Controlling Colorado Potato Beetle (*Leptinotarsa decemlineata*) Populations Under Arid Conditions

**DOI:** 10.3390/insects16020176

**Published:** 2025-02-07

**Authors:** Rauza Abdukerim, Meiramgul Mussina, Gaukartas Abysheva, Nagima Tumenbayeva, Bekzat Mombayeva, Assel Karabayeva, Nurgul Amangeldi, Zamzagul Amangeldikyzy

**Affiliations:** 1 Al-Farabi Kazakh National University, Almaty 050040, Kazakhstan; rauza91@mail.ru; 2 Zhangir Khan West Kazakhstan Agrarian and Technical University, Uralsk 090009, Kazakhstan; meyramgul_70@mail.ru; 3 S. Seifullin Kazakh Agrotechnical Research University, Astana 010000, Kazakhstan; nagi_kosi@mail.ru; 4 Taraz University named after M.Kh.Dulaty, Taraz 080012, Kazakhstan; bekzat.mombaeva.79@mail.ru (B.M.); assel.8485@mail.ru (A.K.)

**Keywords:** arid conditions, *Beauveria bassiana*, *Beauveria pseudobassiana*, biological plant protection, Colorado potato beetle, climate change, entomopathogenic fungi, virulence

## Abstract

Environmentally sound insect pest management strategies require continuous isolation and identification of effective biocontrol agents from different ecosystems. The quest for fungal isolates that are adapted to high temperatures is particularly significant, as they hold the potential to produce mycoinsecticides that are highly effective in semiarid and arid areas. This study aimed to collect new virulent isolates of entomopathogenic fungi (EPF) from Kazakhstan that show promise for controlling Colorado potato beetle (CPB) populations under arid conditions. Local isolates could be adapted to abiotic and biotic factors in the environment in which they survive. The findings of this study should provide insights into the species of EPF that inhabit a specific arid region of Kazakhstan, examining their traits in the regional climate, soil composition, and biological diversity. Throughout 2023–2024, a series of soil samples was collected from diverse locations within the Turkestan region of southern Kazakhstan for the isolation of EPF. The isolation of EPF was conducted using susceptible larvae of *Tenebrio molitor* (Coleoptera: Tenebrionidae), a host that does not have specificity for particular species of EPF. Using this insect bait, 41 pure cultures of EPF were isolated from soils in the arid zone of Kazakhstan. Polymerase chain reaction analysis based on the *TEF1*-α locus showed that all the isolates were indistinguishable from *Beauveria bassiana* ARSEF 2860 species. However, analysis of the ITS locus revealed two cryptic species: *B. bassiana* and *B. pseudobassiana*. For laboratory evaluation of biological activity EPF, the second and third instars of CPB were collected from potato fields in southern Kazakhstan and placed in plastic containers. Test CPB larvae were treated with conidia suspensions at 1 × 10^7^ spores/mL and survival was recorded daily for 3 weeks. The results of the bioassays revealed that all the new Kazakhstan isolates were pathogenic to larvae CPB and caused complete mortality by the end of the 11-day. These results showed that new isolates of EPF were highly virulent against larvae CPB.

## Simple Summary

The Colorado potato beetle (CPB) is a highly damaging pest of potatoes and other plants of the family Solanaceae when its main food source is unavailable. Climate change can lead to increased pressure from CPB in most potato-growing areas and increase the number of generations per year across its range. These impacts highlight the importance of developing integrated and sustainable pest control strategies in the context of climate change. Insecticides are becoming less effective due to the emergence of resistant populations. An ecological alternative to synthetic insecticides is entomopathogenic fungi (EPF), which offers a sustainable and environmentally friendly approach to controlling pest populations. This study investigated the potential of fungi in the genus *Beauveria* to control CPB. The novelty of this study is that effective isolates of the fungus were isolated from the soil in arid zones. The results of the bioassays revealed that all new isolates from Kazakhstan were pathogenic to CPB larvae and could cause complete mortality by the end of the 11-day experiment. This study highlights the importance of searching for new isolates of EPF to control damaging pests adapted to arid zones.

## 1. Introduction

Colorado potato beetle (CPB) is an insect pest that can substantially reduce the potato harvest if left uncontrolled [1]. CPB was first described in 1824 and initially named the nightshade leaf beetle. It now occupies a special place among the phytophagous insects that damage cultivated plants [2]. It is one of the most striking examples of zoological species that have thrived in artificial ecosystems, illustrating the biological features of harmful insect phytophages under conditions of anthropogenic agroecosystem transformation [3]. This beetle became widely known primarily due to its “ecological explosion”, with an unprecedented territorial expansion in both rate and scale. This began in the mid-19th century when the previously harmless leaf beetle shifted from wild thorny nightshades to cultivated potatoes [4]. It causes significant damage to potatoes and eggplants (*Solanum* spp.) and, to a lesser extent, tomatoes (*Solanum lycopersicum*), which are primarily consumed by adults. The beetle is also potentially damaging to pepper, physalis, melon pear, and tobacco crops [5].

The evident adaptation of this beetle for potatoes over other nightshade crops results in an increased concentration of pests in potato fields, higher female fertility when feeding on potato leaves, and higher larval survival rates during development. As a result, the pest remains a major problem for potato farming in many countries, including Kazakhstan [6].

By the early 1990s, CPB had spread throughout Kazakhstan and had become the most damaging pest of potato crops. Since then, many areas of the country have seen massive CPB outbreaks, leading to catastrophic potato crop losses and making protection of potato crops from CPB a matter of national importance [7].

Future management of CPB needs to consider the impacts of global climate change and the availability of host plants on its potential global distribution [8]. High temperature under climate change is one of the main factors that affect the growth and fecundity of CPB [9]. Climate change can lead to increased pressure from CPB in most potato-growing areas and increases in voltinism across the range [10]. These impacts highlight the importance of developing integrated and sustainable pest control strategies.

CPB is mostly controlled by using chemical and agrotechnical methods [11]. The list of insecticides approved for use on potatoes for CPB control is quite extensive, but its rapid development of resistance to synthetic insecticides elevates the challenge of developing anti-resistance protection strategies [4]. The negative impact of pesticides on the biosphere and the need to reduce chemical usage in agriculture has led to the search for alternative pest control methods [12]. The most effective alternative to chemical pesticides is natural agents (entomophagous and microorganisms), which are inherently safe for humans and the environment. Compared to other biological agents, entomopathogenic fungi (EPF) have the greatest potential as they cause severe epizootics in insects, more so than bacteria and viruses [13]. Biological control of insect pests with EPF is one of the most desirable and effectual practices involving natural isolates, which restrict the activity of pests and can be used as an alternative to synthetic insecticides. EPF have several biological attributes, such as target-specificity, high reproductively, short generation time, and long survival, which are significant for the biocontrol of insect pests [14].

Currently, there are more than 750 recognized species of EPF across various genera worldwide, many of which have evolved to coexist with insects [15]. EPF are presently positioned in four legitimate phyla (Ascomycota, Basidiomycota, Chytridiomycota, and Zygomycota) and are broadly distributed in various natural ecosystems [14,16]. Most parasitic species among these belong to various genera in the order Hypocreales [17].

EPF have been used to economically control important pests for nearly 200 years, and today there are over 100 EPF mycoinsecticide products available [18]. However, EPF as a total share of biopesticides on the global pesticide market does not exceed 6%, which is significantly lower than the share of synthetic insecticides [19]. A closer investigation of the share of the overall industry of biopesticides has uncovered that mycoinsecticides have contributed to an exceptionally small proportion of the market of biopesticides because a large proportion are formulations based on the bacterium *Bacillus thuringiensis*. The main explanation for mycoinsecticides being only a small proportion of the overall market is their moderately slow kill rate and an expansion in the market value share would legitimately respond to improved killing speed [20]. However, with the wider recognition of the potential negative effects of synthetic pesticides on nature, increasing development of insecticide resistance, and expanding developing enthusiasm for so-called organic/natural food, there is now a greater opportunity for successful marketing of EPF mycoinsecticides [14].

The EPF genus *Beauveria*, which infects a wide range of insects and is widely distributed, has significant potential in field applications [21,22]. This is because most modern mycoinsecticides are based on these fungi, in particular, *B. bassiana* and *B. brongniartii* [23].

The genus *Beauveria* belongs to the phylum Ascomycota, class Sordariomycetes, order Hypocreales, family Cordycipitaceae [17,24]. The genus comprises over 28 species [24,25,26,27,28]. The genus *Beauveria* consists of a complex array of cryptic species waiting to be elucidated [29]. Cryptic sibling species are often specialized for different hosts; therefore, it is essential to elucidate their genetic and species status [24,25,26,27,28,29]. Taxonomy is essential for understanding the relationship between specific species and their geographic origin. Such precise delimitation enlarges the potential for finding suitable biocontrol agents and their safe use. In the context of extensive global research, only a few studies on EPF have been conducted in Kazakhstan. A comprehensive understanding of the diversity among EPF is crucial for discovering promising isolates that can effectively combat harmful pests. In this regard, this study aimed to collect virulent EPF isolates from Kazakhstan with the potential to control CPB populations in the arid zones of southern Kazakhstan.

## 2. Materials and Methods

### 2.1. Sample Collection and Fungal Isolation

**Figure 1:**
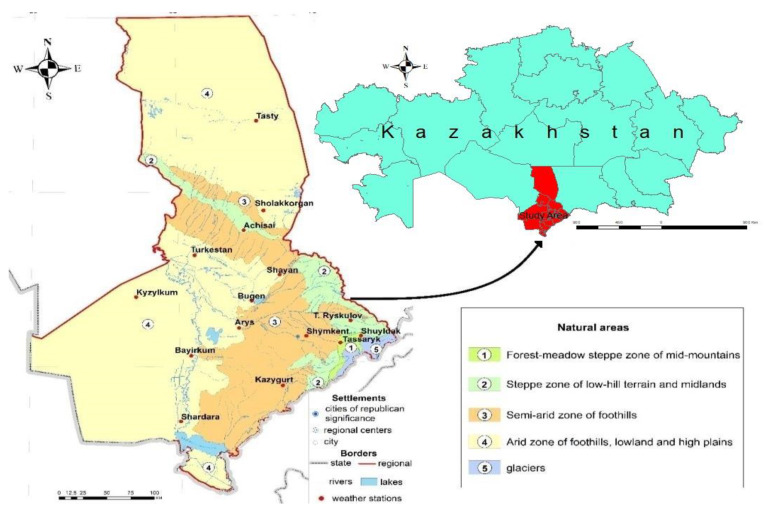
Geographic location of the study area [31].

EPF in the genus *Beauveria* are widespread in natural ecosystems, including soil [30]. From spring to autumn 2023–2024, soil samples were collected from various locations (agroecosystems) in southern Kazakhstan (Turkestan region) (N41.58289 E068.79088; N41.565 E069.12486; N41.662382 E068.332032; N41.95229 E068.23202; N41.72442 E068.19060; N41.26057 E068.24502; N43.52712 E068.02665; N42.91714 E069.11839; N41.897699 E068.999699; N41.894016 E069.000387; N42.135229 E069.011047; N42.01118 E069.89464; and others) using a soil probe to a depth of 20 cm and placed in sterile ziplock bags. Located at the geographical coordinates of 41–46°N and 65–74°E, the region features four main natural zones (Figure 1): a forest-meadow steppe zone in the mid-mountains; a steppe zone across low-hill terrain and midlands; a semiarid zone in the foothills; and an arid zone that spans lowland and high plains. These zones are characterized by a variety of climatic conditions. The relief of the territory is mostly flat with a general slope to the southwest. The region examined was within the arid zone, distinguished by its marked continentality, lack of moisture, and high temperatures. High continentality manifests in sharp temperature contrasts between day and night, winter and summer. Aridity is one of the main distinguishing features of the regional climate [31]. The soil cover of the study area was represented by ordinary sierozem, light chestnut soils, and Calcisols [32,33]. The environmental and climatic factors in southern Kazakhstan create favorable conditions for the year-round growth of numerous crops, both in the field and in greenhouses. Therefore, there is a focus on growing vegetables, melons, and industrial crops in this region.

EPF were isolated using susceptible yellow mealworm larvae, *Tenebrio molitor* (Coleoptera: Tenebrionidae), which does not have specificity for any particular EPF species [34]. The collected soil was ground and sieved. It was then stored at 4 °C for further analysis. From each soil sample, 250 g was placed in clean plastic containers with air holes in the lids (5 replicates for each sample). Five *T. molitor* larvae were added per container along with food (sterile oat bran) to prevent premature death without infection. The containers were incubated in the dark at 25 °C and inverted during the first week [34]. Every 3–4 days, the larvae were examined for signs of mycosis, which would confirm infection. The surface of dead bait larvae was sterilized with 1% sodium hypochlorite and these were then placed in a moist chamber (at 25 °C for 7 days). When signs of mycosis appeared, conidia were collected from the surface of the larvae and transferred to Sabouraud dextrose agar for further growth. Subculturing was performed as needed until a pure culture was obtained. The fungi samples were incubated in a thermostat at 25 °C for further growth.

### 2.2. Molecular Identification of the Species Composition of Entomopathogenic Fungi

Extraction of DNA was performed by the cetyltrimethylammonium bromide (CTAB) method. After homogenization, samples were incubated for 1 h at 65 °C in CTAB buffer with 100 mM TrisHCl, 1.4 mM NaCl, 1.4 mM EDTA, 4% CTAB, 2% polyvinylpyrrolidoneand, and 0.2% β-mercaptoethanol. Following incubation, the mix was centrifuged for 5 min at 5000 rpm to remove debris. Then, 1 mL of a chloroform/isoamyl alcohol mixture (24:1) was added and the mix was centrifuged at 13,000 rpm for 12 min. The supernatant was transferred to fresh tubes, followed by the addition of isopropanol to make up two-thirds of the final volume. This mix was then cooled to −20 °C for 1 h and DNA precipitation was performed by centrifuging at 13,000 rpm for 15 min. The supernatant was then removed, and the DNA precipitate was washed in 70% ethanol followed by precipitation at 13,000 rpm for 15 min. The DNA precipitate was dissolved in 40 μL of water and incubated with RNAse A (Thermo Scientific, Waltham, MS, USA) at 37 °C for 1 h. DNA quality was assessed by electrophoresis in 1.5% agarose gel in a chamber with TAE buffer at 400 mA and 70 V for 30 min, and DNA was analyzed under UV light. The purity and concentration of DNA were also checked on a NanoDrop One spectrophotometer (Thermo Scientific).

For the sequencing and subsequent analysis of the ITS region, polymerase chain reaction (PCR) with primers internal transcribed spacer ITS5 (59-GGAAGTAAAAGTCGTAACAAGG) and ITS4 (59-TCCTCCGCTTATTATTGATATATATGC) was performed. The resulting amplified region length was 593 bp. *TEF1*-α (translation elongation factor 1-alpha) gene was amplified using primers 983F (59-GCYCCYGGHCAYCGTGAYTTYAT) and 2218R (59-ATGACACACCRACRGCRACRGCRACRGTYTG). Consequently, reaction products of 1000 bp were produced, encompassing the region of the elongation factor. The reaction mixture contained: 1X Standard Taq Reaction Buffer (New England BioLabs Inc., Ipswich, MS, USA), 0.2 µM dNTP, 0.2 µM of each primer pair, and 1 unit of Taq-polymerase (New England BioLabs Inc., 5000 U/mL). Then, 50 ng of extracted DNA was used for amplification according to the following protocol: denaturation at 94 °C for 2 min, followed by 40 cycles (denaturation at 94 °C for 30 s; primer annealing at 54 °C for 1 min; chain elongation at 72 °C for 1 min) for ITS, and 45 cycles (denaturation at 94 °C for 30 s; primer annealing at 54 °C for 1 min; and chain elongation at 72 °C for 1 min 30 s) for *TEF1*-α, followed by 10 and 15 min final elongation at 72 °C, respectively. Electrophoresis was performed on the amplification products in a 1.5% agarose gel and the gel was subsequently stained with ethidium bromide for analysis.

Sequencing of target loci was performed on a MinION Mk1B portable nanopore sequencing device (ID MN43770) from Oxford Nanopore Technologies on FLO-MIN114-type flow cells. The library was developed in accordance with the SQK-RBK114.96 protocol of the Rapid Barcoding V14 kit. Concentration was measured with a Qubit Flex Fluorometer using the Qubit 1X dsDNA HS assay Kit according to the manufacturer’s protocol. Basecalling (and subsequent trimming of barcodes) was conducted using Dorado (v 7.4.12) basecaller on MinKNOW (v 24.06.8) software. The data obtained were analyzed on Geneious Prime^®^ 2024.0.5. The database of “Fungi: ITS project” from NCBI was used as reference sequences for mapping with the Minimap2 plugin (v 2.2.0). As for *TEF1*-α, the database was established by downloading approximately 8K sequences from NCBI using “TEF1-α”, “alpha” keywords, and filtering down to fungi species. Based on generated consensus sequences of ITS and *TEF1*-α, phylogenetic trees were constructed using the program MEGA11 (v 11.0.13) with a maximum likelihood method.

### 2.3. Preparation of the Working Suspension and Determination of Titer Using the Goryaev Chamber

To prepare the working suspension, dry fungal biomass was scraped using a scalpel from the Sabouraud dextrose agar medium. The mycelium collected was placed in a glass test tube and covered with 2 mL of sterile water. From this mixture, 1 mL was transferred to a sterile test tube containing 9 mL of sterile water with 0.01% Twin 80, then the suspensions were diluted to a conidial titer of 1 × 10^7^ spores/mL. The resulting suspension was thoroughly mixed until it became cloudy. A drop of the semi-preparative form was applied to the Goryaev chamber, and excess liquid was removed with filter paper. The spore titer in 1 mL of suspension was determined by counting the spores in large squares and calculated as:

$$T=\frac{\sum spore\times 50}{k}\times 5000$$

T—spore titer in spores/mL;

∑ spore—the sum of spores counted in large squares;

k—the number of large squares with counted spores.

As necessary, the suspension was diluted with water to the required concentration, or a new batch was prepared. The prepared solution was used within 1–2 h, as further germination of fungal conidia reduces the effectiveness of the inoculum [35].

### 2.4. Laboratory Evaluation of Biological Activity

The second and third instars of CPB were collected from southern Kazakhstan potato fields in the districts of Kazygurt, Keles, Maktaaral, Sairam, Saryagash, Tolebi, and Tulkubas.

To determine the biological activity of the EPF against CPB larvae, test larvae without visible damage were first transferred to a Petri dish and sprayed from 35 cm with a handheld sprayer, using 2 mL of conidial suspension of the test isolate. All isolates were tested with a suspension of 1 × 10^7^ conidia/mL using this spray method. After the treated larvae were carefully transferred with a brush to a new container containing clean potato leaves. They were placed in a 1 L plastic container covered with milling gauze with 5 individuals per cage. The larvae were inoculated at room temperature. The experiments were repeated four times. Control groups were treated with distilled water to create identical conditions for all insects. They were kept under laboratory conditions at 25 °C. All containers were inspected daily for 20 days after treatment and dead individuals were collected. Surviving larvae were fed on untreated leaves for the rest of the experiment. The cadavers were then placed on slides in a moist chamber to determine the cause of death and the level of fungal colonization.

Biological effectiveness was calculated using Abbott’s formula [36]:

$$C=\frac{100(A-B)}{A}$$

C—percentage of pest mortality;

A—average number of individuals before treatment;

B—average number of individuals after treatment.

### 2.5. Statistical Analysis

Statistical analysis was performed using the analysis of variance method using Microsoft Office Excel spreadsheets, IBM statistical application package, and Survival Library version 3.1-12 Therneau (package for survival analysis in R). Survival data were recorded for larvae treated with different isolates over time, including the number of dead and alive individuals. Survival probabilities and ST50 were estimated using Kaplan–Meier curves.

## 3. Results

### 3.1. Species Composition of Entomopathogenic Fungi

To isolate entomopathogenic micromycetes, more than 150 soil samples were collected from various points of agrocenoses in the focus region. In laboratory conditions, larvae of *T. molitor* were placed in these soil samples as bait for EPF. After 29 days, 647 of 865 larvae died. Signs of mycosis appeared in more than 60 larvae of the 647 dead larvae. In total, 41 isolates of EPF were obtained in pure culture.

A morphological analysis of the species composition of the isolated EPF indicated that all isolates had features typical of the genus *Beauveria*. These isolates are characterized by colonies that are either white or beige, with a darker hue at the center, and fluffy or woolly and white mycelium.

**Figure 2:**
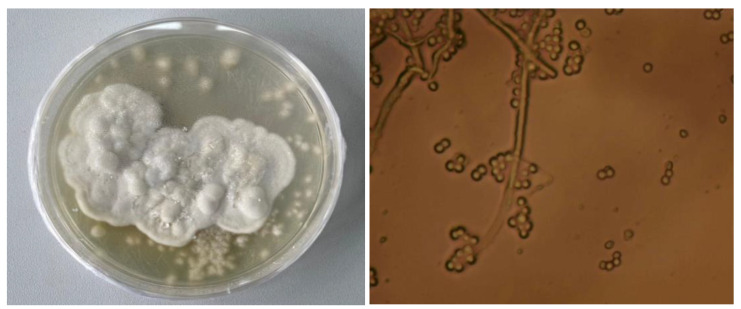
Colonial morphology and spores of isolates fungi genus *Beauveria*.

To determine if there were cryptic species of *Beauveria*, an analysis of the conidial size from the isolates was carried out (Figure 2). However, no significant differences in spore size in these isolates were found. However, these characters are largely concordant among *Beauveria* species, making it difficult to distinguish them morphologically alone. Therefore, to clarify the taxonomic status of the natural isolates, PCR analysis was performed on the DNA region ITS and *TEF1*-α, as both are routinely used in fungal taxonomy.

**Figure 3:**
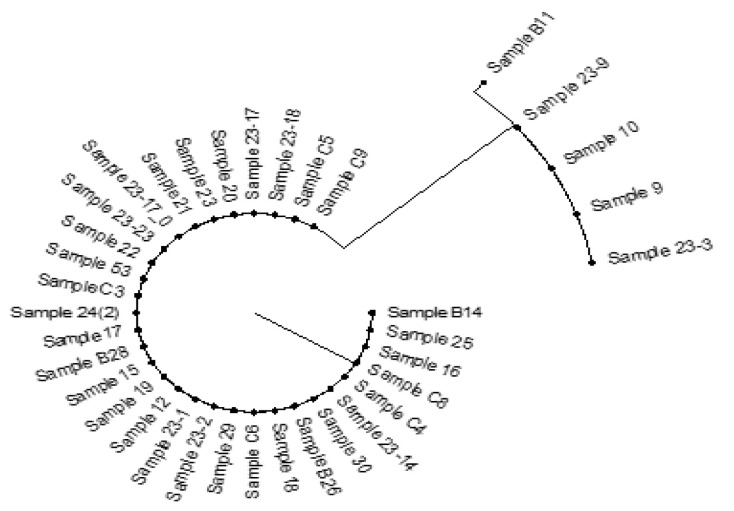
Phylogenetic analysis of ITS regions.

In the examination of fungal taxonomy, 41 isolates were sequenced resulting in the identification of 36 isolates. The primary regions of interest for various nanopore-based high-throughput sequencing techniques sequencing were the ITS and *TEF1*-α target sites. PCR analysis of the DNA region *TEF1*-α revealed that all isolates belong to the species *B. bassiana* ARSEF 2860. However, the analysis of the ITS region identified the presence of two cryptic species: *B. bassiana* and *B. pseudobassiana*. Isolates sequencing took an average of 6 h and provided sufficient information for further processing. Sequencing of isolates in total yielded 898.71 thousand reads and 502.62 Mb of nucleic data. A total of 10 to 20 thousand reads were produced for each isolate. The results from mapping to reference sequences are as follows: for the ITS regions, all isolates belonged to the species *B. bassiana* in 84% of cases, i.e., for 31 isolates. Between 55% and 83% of all mapped reads belonged to isolate ARSEF 1564 (NR_111594.1). The remaining five isolates were aligned to *B. pseudobassiana* of isolate ARSEF 3405 (NR_111598.1), as shown in Figure 3.

**Figure 4:**
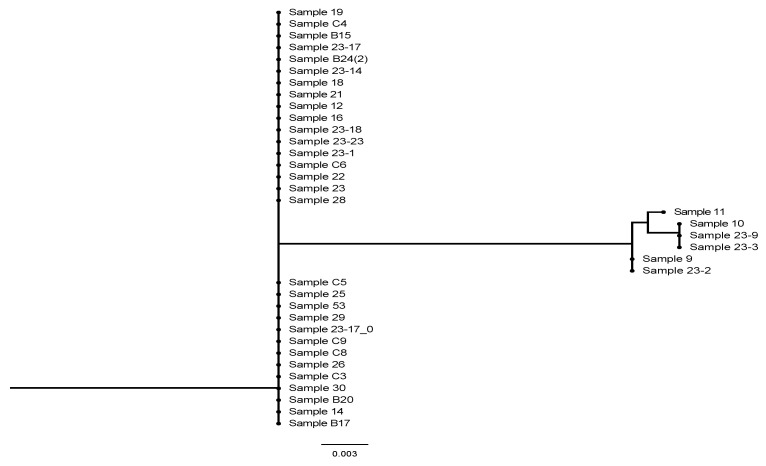
Phylogenetic analysis of *TEF1*-α (translation elongation factor 1-alpha) gene regions.

In addition, isolate B11 had the highest level of genetic diversity, recording an evolutionary distance of 0.03. The subsequent isolates in terms of genetic diversity were 23-9, 10, 9, and 23-3. The other isolates had genetic homology. In this study, sequences obtained from the PCR fragment using the 983F-2218R primers, covering the last two-thirds of the gene, were used to construct a phylogenetic tree for the *TEF1*-α gene. This region of *TEF1*-α is intron-free in the Hypocreales and, apart from a unique 15 base pair insertion (five codons) found in the outgroup *Paecilomyces farinosus*, had collinearity in *Beauveria* (Figure 4). All isolates were successfully amplified with the 983F-2218R markers. Mapping showed unambiguous results in all cases.

Notably, there was a distinct clade consisting of isolates 23-2, 9, 11, 10, 23-9, and 23-3, indicating some genetic dissimilarity of some isolates in one species. The observation that isolate B11 had the highest genetic diversity of the isolates indicates a potentially unique ecological niche or evolutionary pathway. Also, the clustering of several specimens (specifically 23-2, 9, 11, 10, 23-9, and 23-3) into a separate clade indicates notable genetic dissimilarity. This genetic divergence could point to microevolutionary processes or local adaptations within *B. bassiana* populations. Importantly, isolates 11, 10, 23-9, 23-3, and 9 make a distinctive clade both in phylogenetic trees of ITS and *TEF1*-α loci. Several factors could account for the genetic differences identified in the separate clade of *B. bassiana* isolates, including local adaptations to specific ecological niches, which arise from the collection of isolates in geographically distinct locations. This can be influenced by a range of environmental conditions, including soil type, temperature, and humidity. To summarize, the differences in genetic makeup among the *B. bassiana* isolates could reflect local adaptations and microevolutionary processes including genetic drift, mutation, and natural selection. These findings indicate that all isolates were indistinguishable from *B. bassiana* ARSEF 2860. However, the analysis of the ITS region identified the presence of two cryptic species: *B. bassiana* and *B. pseudobassiana*.

### 3.2. Assessment of the Virulence of Natural Entomopathogenic Fungal Isolates Against CPB Larvae

An important aspect of evaluating the potential of producer isolates is assessing their biological activity. Observations showed significant variability in virulence among the studied isolates (Table 1). The mortality of CPB larvae on the fifth day after treatment varied from 30% to 50%. The mortality only increased in the subsequent days, and by day 7, it had reached a maximum of 79%.

**Figure 5:**
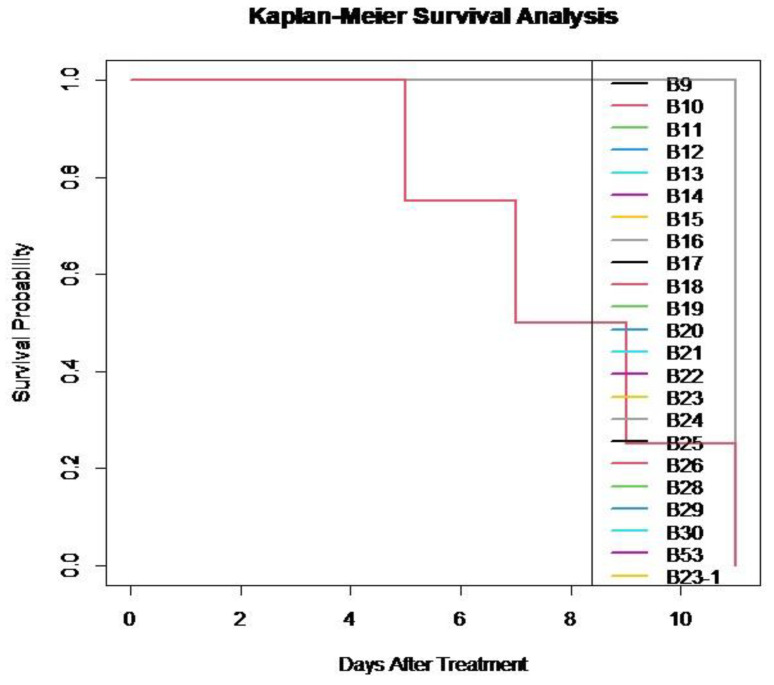
Survival probabilities of larvae *Leptinotarsa decemlineata* treated by fungal suspensions.

Most isolates had an ST50 of 8 days, indicating faster mortality compared to the control group. Most isolates had significantly reduced survival times (ST50 of 8 days) compared to the control group (Figure 5). These results indicate that the isolates effectively reduce larvae survival within the observed time frame. The log-rank test confirmed that survival differences between groups are statistically significant.

The highest mortality was observed with isolates B13, B14, B15, B19, B21, B22, B23, B24, B25, B53, B23-23, Bc4, Bc5, Bc7, and Bc8. The mortality after infection with these cultures reached 80% to 90% by day 7 and up to 100% by day 11, while for other isolates, the mortality at those times varied from 60% to 70% and up to 80% to 90%, respectively. The results indicate the high effectiveness of these isolates.

## 4. Discussion

EPF are regarded as promising biocontrol agents for many important pests of agriculture around the world [37]. The use of fungal pathogens provides numerous advantages: high yield, cost-effectiveness, preservation of beneficial organisms, safety to humans, no harmful effect on the environment, and varied biodiversity [38,39]. Environmentally sound insect pest management strategies require continuous isolation and identification of effective biocontrol agents from a range of ecosystems [38]. Consequently, it is important to obtain locally adapted biological control agents because such isolates may be more effective than non-local isolates as well as being more cost-effective [40]. Local isolates are likely to be adapted to abiotic and biotic factors in the environment in which they survive [41]. In this regard, we collected isolates of EPF from Kazakhstan that offer promise for controlling CPB populations under the arid zones of southern Kazakhstan.

Understanding the habitat adaptations of EPF is critical to improving the efficacy, persistence, and cost of these fungi as microbial insecticides [42]. Previous studies of EPF have selected EPF for field release based solely on their efficacy in laboratory bioassays without consideration of their microhabitat adaption and ecological constraints. Bidochka assumed that EPF population genetics are closely related to host insects [43]. However, recent research revealed that *B. bassiana* has adapted to selected habitats, and any evidence of an insect–host-related population structure should be viewed primarily as coincidental and not as a result of coevolution [28,34,44,45].

In this study, EPF of genus *Beauveria* were isolated from soils of southern Kazakhstan (Turkestan region). The results support the notion that EPF are common members of the soil microbiota [46]. Soil is the natural reservoir of most EPF, which protects them from damaging solar radiation and extreme temperatures and provides a medium for enhanced persistence, dispersal, and growth [44,46]. Soil is the most commonly examined environment for assessing *Beauveria* diversity [47]. Therefore, in 2023–2024, a series of soil samples were collected from diverse locations within the Turkestan region of southern Kazakhstan to facilitate the isolation of entomopathogenic fungi. There are two methods for isolating entomopathogenic fungi: selective media and insect baits [48,49]. A significant barrier to the application of the selective media isolation method is the relatively slow growth of hypocrealean EPF when contrasted with the prevalent opportunistic saprotrophic fungi found in the soil environment [34]. The more effective method involves using susceptible larvae of *Galleria mellonella* (Lepidoptera: Pyralidae) or *T. molitor* [34,50]. Keyser with colleagues claim the number of EPF detected in soil can be affected by the methods of isolation [51]. However, Keller determined the rate of detection of EPF in soil by either method was similar [52]. In this study, the isolation of EPF was conducted using susceptible larvae of *T. molitor*, which does not have specificity for any particular species of EPF [34]. In total, 41 pure isolates of EPF were obtained.

The classification of EPF has generally relied on their respective ultrastructure and morphology as the established criteria. The morphological analysis of the species composition of the isolated EPF indicated that all isolates had characteristics consistent with the genus *Beauveria* [53]. However, no significant differences in spore size in these isolates were found, making it difficult to distinguish them morphologically alone. DNA-based molecular characterization is a powerful tool for the more precise arrangement of EPF into taxa [54]. In this regard, isolates were genetically characterized using the DNA region ITS and *TEF1*-α gene.

ITS primers are used to reproduce the ITS region, which is one of the general diagnosis primers in fungi, as it is widely used in the diagnosis of EPF [55,56]. In this regard, it was used to identify fungi isolated in this study. As a result, the phylogenetic placement of these isolates demonstrated that they had similar evolutionary homology with other isolates *B. bassiana* and *B. pseudobassiana* from the NCBI database. The ITS region in various fungi has routinely been sequenced for identification and phylogenetic studies [57]. However, this general marker is not sufficient to capture the species diversity of EPF. The results of sequence locus *TEF1*-α of EPF provide a more informative diagnosis [42,58]. Both the ITS and *TEF1*-α regions are often used for fungal taxonomy. Rehner and Buckley used ITS and *TEF1*-α loci to delineate new terminal lineages that are phylogenetically distinct from but morphologically similar to *B. bassiana* [25]. Rehner used *TEF1*-α to revise the systematics of the anamorphic, entomopathogenic genus *Beauveria* [27]. Their research concluded that *B. bassiana* contains an unknown number of cryptic lineages, several of which are distributed across continents and manifest as multispecies assemblages in various natural and agricultural environments. The ITS region is highly variable among fungal species and the *TEF1*-α gene being a more conserved region provides additional resolution. In this regard, PCR analysis was conducted on the *TEF1*-α loci to clarify the taxonomic status of natural isolates. PCR analysis of the nuclear DNA *TEF1*-α locus showed that all isolates belong to the species *B. bassiana*. Strengthening both elements not only broadens the range of genetic diversity assessed but also enhances the accuracy of species identification using multiple markers.

Only the fungal genus identified in the soil of the study area was *Beauveria*, which might be due to the absence of diverse insect hosts, high temperatures, soil composition, or use of chemical pesticides in farmland. No other species of the EPF were identified, perhaps due to their distribution being limited by specific environmental conditions or insect hosts [31,41,47]. Other studies of regions with a temperate climate and fertile soil have shown a more diverse EPF species composition. They concluded that the variation in the occurrence of EPF from soil samples can be explained based on the influence of biotic and abiotic factors including availability of insect hosts, temperature, humidity, UV-radiation, soil type, and organic matter [59]. Also, previous studies determined that the genus *Beauveria* was the dominant species in the soil samples [49]. Wakil report a greater abundance and distribution of *Beauveria* spp. (10 species representing ~6%), followed by *Metarhizium* spp. (5 species), in 168 soil samples collected from different cultivated and uncultivated sites [60]. In a study by Korosi of the 240 soil samples, 26% contained *Beauveria* spp. and 33% *Metarhizium* spp. [46]. Sharma also identified a slightly higher incidence (27%) of *B. bassiana* among various EPF isolated from 183 soil samples using an insect bait method [59]. Our results reinforce these conclusions, indicating that *B. bassiana* was the dominant species identified in the soil collected from the Turkestan region of southern Kazakhstan. A possible reason for the higher occurrence of *B. bassiana* is that it is capable of surviving in and adapting to diverse ecological niches through the activation of distinct gene sets [61]. Also, it might be because the population of *B. bassiana* was associated with agricultural areas and *B. pseudobassiana* was more strongly associated with forest habitats [30]. However, Castro-Vásquez report a low correlation between geographical origin and variability among the fungi genus *Beauveria* [62]. Further in-depth investigations are required to understand the impact of environmental factors on the distribution patterns and preferences of entomopathogenic fungi.

Literature analysis revealed that many studies have been conducted to determine the effects of different *B. bassiana* isolates on the adult, larval and pupal stages, and hatching rates of CPB [63]. Numerous studies were based on the periodical introduction of new *B. bassiana* isolates and showed that these could potentially reduce the incidence of insecticide resistance [64]. The findings of our research differ from the previous studies that compared the efficacy of EPF isolates on different stages of CPB [65,66,67]. According to a study by Wraight and Ramos, *B. bassiana* is more virulent against earlier versus later instars of CPB [68]. The reason for this could be lower efficacy against adult beetles due to the hardening of the insect cuticle as it develops from larva to adult. A study by Charnley determined that the mortality of insects due to *B. bassiana* infection in the late-stage instars was low due to the hardening of the insect cuticle [69]. Akbarian also found that the susceptibility of second instars to all *B. bassiana* was significantly higher than the fourth instars of CPB [64]. Therefore, in our study, the isolates of *B. bassiana* were tested on the second and third instars of CPB. If these isolates did not have virulence against susceptible instars, they would not be effective against adults. In our experiments using larval stages, it was determined that the mortality due to all EPF isolates in the genus *Beauveria* were significantly greater than the mortality in the control. These isolates were obtained from soils in southern Kazakhstan and exhibited high virulence against CPB, with mortality rates ranging from 90% to 100% by 11 days after treatment. The observed variation in virulence of different isolates highlights the importance of selecting appropriate isolates for further studies. An analogous result was found by Shafighi with two *B. bassiana* isolates giving 60% mortality of second-stage larvae within 15 days [70]. Also, Ozturk reported that the isolates they tested caused mortalities between 57% and 100% in young (second and third instar) larvae, between 37% and 100% in fourth instars, and between 23% and 86% in 1-week old adults; 3 and 7 days after treatment [66]. Polat determined that the mortality of adult insects caused by 33 different fungal isolates tested on days 7 and 11 was lower than the mortality of the third instars on days 5 and 7 [71]. An earlier study by Baki determined that the virulence of tested isolates was variable with mortality increasing with exposure time [72]. Such contrasting results might be due to the geographical origin of the EPF tested or other factors such as temperature and humidity [73,74]. Unlike other bio agents, *B. bassiana* represents an efficient control against adults and all larval stages of CPB and it can continue to grow after the application. This offers the potential for a high degree of control during the potato-growing season. However, the most notable limitation of *B. bassiana* seems to be its vulnerability to high temperatures and drought [68]. Therefore, the isolation of EPF from arid zones is important as these isolates are likely to be adapted to abiotic factors in the environment in which they survive.

A common issue in scientific studies is whether the virulence of EPF depends on the source of isolation or geographic origin. Some authors suggest that the host species from which the EPF were isolated is more important than their geographic origin [75]. They reported that EPF isolated from CPB populations is more virulent and promising for pest control compared to isolates from other sources. Conversely, other authors argue that despite the source of isolation, other entomopathogenic fungal isolates can also be lethal and thereby considered promising for CPB control [70]. Our results support the latter view and demonstrate that isolates of EPF are highly virulent against CPB regardless of the source of isolation.

## 5. Conclusions

The range of EPF present in the soils of southern Kazakhstan, particularly in the Turkestan region, is notably modest and sparse, reflecting the regionally specific soil and climatic characteristics. Only fungi of the genus *Beauveria* were isolated, which are considered cosmopolitan. Isolation of viable EPF demonstrates adaptation to arid conditions, which is important in the context of climate change.

As a result of using insect bait, 41 isolates of EPF were obtained in pure culture from soils in the arid zone of Kazakhstan with 36 isolates fully identified. The assessment of species composition in the isolated cultures, focusing on morphological features, demonstrated that the majority of the EPF isolates are in the genus *Beauveria*. Standard DNA loci for identification of EPF *TEF1*-α and ITS were used for molecular analysis. PCR analysis of the DNA region *TEF1*-α revealed that all isolated isolates were indistinguishable from *B. bassiana* ARSEF 2860. However, the analysis of the ITS region identified two cryptic species: *B. bassiana* and *B. pseudobassiana*.

Most isolates of EPF exhibited high virulence against CPB, with mortality rates ranging from 90% to 100% 11 days after treatment. The most virulent isolates were B13, B14, B15, B19, B21, B22, B23, B24, B25, B53, B23-23, Bc4, Bc5, Bc7, and Bc8, and are considered the most promising for use in arid zones.

## Figures and Tables

**Table 1:** Corrected mortality, mean survival time (ST50), and lethal concentration (LC50) of larvae *Leptinotarsa decemlineata* treated by fungal suspensions.

№	Isolates	LC50	LC90
1	B9	9.13	16.5
2	B10	6.67	12.1
3	B11	8.37	15.2
4	B12	6.23	11.3
5	B13	4.58	8.37
6	B14	5.48	9.97
7	B15	4.86	8.86
8	B16	7.73	14.0
9	B17	6.67	12.1
10	B18	6.23	11.3
11	B19	4.58	8.37
12	B20	5.48	9.97
13	B21	5.15	9.39
14	B22	4.58	8.37
15	B23	4.58	8.37
16	B24	4.58	8.37
17	B25	4.86	8.86
18	B26	7.73	14.0
19	B28	5.48	9.97
20	B29	6.23	11.3
21	B30	4.86	8.86
22	B53	4.58	8.37
23	B23-1	8.37	15.2
24	B23-2	7.16	13.0
25	B23-3	5.83	10.6
26	B23-9	13.9	25.2
27	B23-11	7.16	13.0
28	B23-13	7.73	14.0
29	B23-27-0	6.23	11.3
30	B23-14	8.37	15.2
31	B23-17	5.15	9.39
32	B23-18	7.73	14.0
33	B23-23	5.15	9.39
34	B23-25	7.16	13.0
35	Bc3	7.73	14.0
36	Bc4	4.33	7.92
37	Bc5	5.83	10.6
38	Bc6	9.13	16.5
39	Bc7	6.23	11.3
40	Bc8	6.23	11.3
41	Bc9	6.23	11.3

## Data Availability

All data are presented in this article.
